# DNA topoisomerase II**α** and -**β** expression in human ovarian cancer

**DOI:** 10.1038/sj.bjc.6690120

**Published:** 1999-02

**Authors:** S Withoff, A G J van der Zee, S de Jong, H Hollema, E F Smit, N H Mulder, E G E de Vries

**Affiliations:** Division of Medical Oncology, University Hospital Groningen, Groningen, The Netherlands; Division of Gynaecologic Oncology, University Hospital Groningen, Groningen, The Netherlands; Department of Pathology, University Hospital Groningen, Groningen, The Netherlands; Department of Pulmonary Diseases, University Hospital Groningen, Groningen, The Netherlands

**Keywords:** DNA topoisomerase IIα, and -β, ovarian cancer

## Abstract

To study DNA topoisomerase IIα (Topo-IIα) and -β expression and regulation in human ovarian cancer, 15 ovarian tumour samples were investigated. To compare different levels of expression, the samples were screened for topo IIα and -β mRNA with Northern blotting and a quantitative reverse transcriptase polymerase chain reaction (RT-PCR) assay for Topo-IIα mRNA. Additionally, protein levels were determined with Western blotting and topoisomerase II activity levels with the decatenation assay. The results obtained were compared with each other and with the tumour volume index of the samples. In tumours with a tumour volume index ≥ 50%, the mRNA levels (as determined by Northern blotting) and protein levels for each isozyme were in accordance. Additionally, correlations were found between Topo-IIα RT-PCR data and Topo-IIα Northern blot results, and between Topo-IIα RT-PCR data and Topo-IIα protein levels. Interestingly, Topo-IIβ protein levels correlated better with Topo-II activity than Topo-IIα protein levels. In eight ovarian cystadenoma samples, no Topo-IIα protein could be found. In only three out of eight of these cystadenomas, Topo-IIβ protein could be detected. These findings suggest that Topo-IIα and Topo-IIβ protein levels are up-regulated in ovarian cancer and may indicate that Topo-IIβ is an interesting target for chemotherapy in ovarian tumours. © 1999 Cancer Research Campaign


*British Journal of Cancer* (1999) **79**(5/6), 748–753
© 1999 Cancer Research Campaign
Article no. bjoc.1998.0120

### DNA topoisomerase IIα and -β expression in human ovarian cancer

**S Withoff[1], AGJ van der Zee[2], S de Jong[1], H Hollema[3], EF Smit[4], NH Mulder[1] and EGE de Vries[1]**

[1]Division of Medical Oncology, [2]Division of Gynaecologic Oncology, [3]Department of Pathology, and [4]Department of Pulmonary Diseases, University Hospital Groningen, The Netherlands

**Summary** To study DNA topoisomerase IIα (Topo-IIα) and -β expression and regulation in human ovarian cancer, 15 ovarian tumour samples were investigated. To compare different levels of expression, the samples were screened for topo IIα and -β mRNA with Northern blotting and a quantitative reverse transcriptase polymerase chain reaction (RT-PCR) assay for Topo-IIα mRNA. Additionally, protein levels were determined with Western blotting and topoisomerase II activity levels with the decatenation assay. The results obtained were compared with each other and with the tumour volume index of the samples. In tumours with a tumour volume index ≥ 50%, the mRNA levels (as determined by Northern blotting) and protein levels for each isozyme were in accordance. Additionally, correlations were found between Topo-IIα RT-PCR data and Topo-IIα Northern blot results, and between Topo-IIα RT-PCR data and Topo-IIα protein levels. Interestingly, Topo-IIβ protein levels correlated better with Topo-II activity than Topo-IIα protein levels. In eight ovarian cystadenoma samples, no Topo-IIα protein could be found. In only three out of eight of these cystadenomas, Topo-IIβ protein could be detected. These findings suggest that Topo-IIα and Topo-IIβ protein levels are up-regulated in ovarian cancer and may indicate that Topo-IIβ is an interesting target for chemotherapy in ovarian tumours.

**Keywords:** DNA topoisomerase IIα and -β; ovarian cancer

Clinical data in ovarian cancer indicate that resistance to chemotherapeutic drugs in these tumours is both intrinsic and acquired. Taxol, etoposide, cisplatin, anthracyclines and cyclophosphamide are currently used in treatment (Cannistra, 1993; Trimble et al, 1994). The finding of intrinsic or acquired resistance to these drugs, which have different targets, suggests that differences in expression levels of various resistance-associated proteins may be expected in primary ovarian tumours. Moreover, several resistance mechanisms may be triggered when resistance develops during chemotherapeutic treatment (see Van der Zee et al, 1995 for review).

Recently, it was described that DNA topoisomerase II (Topo-II) is involved in drug resistance and sensitivity in human tumours. Two isoforms have been described (Topo-IIα and Topo-IIβ). It was shown that these DNA conformation-controlling nuclear enzymes are the target for several drugs that are widely used in the clinic as anti-cancer chemotherapy (Pommier, 1993). Examples of such drugs are anthracyclines (e.g. doxorubicin) and epipodophyllotoxins (e.g. etoposide). These drugs exert their action by stabilizing a reaction intermediate formed during the catalytic cycle of Topo-II. The presence of this stabilized protein–DNA complex, which is called the cleavable complex, interferes with several processes which take place at DNA level (transcription and replication), causing DNA damage and ultimately cell death. Topo-II-related drug resistance is caused by a decrease in cleavable complex formation in the nucleus, which will lead to less DNA damage and less cell death.

In human ovarian tumours, it was found that Topo-II decatenation activity increased with malignant transformation, and decreased after first-line platinum/cyclophosphamide treatment (Van der Zee et al, 1991, 1994*a*). This decrease was the more remarkable because Topo-II is not the primary target for these drugs. In these studies, no correlation was found between mitotic index and Topo-II activity levels, and no evidence for an altered (mutated) Topo-II was found. Additionally, no relation was found between Topo-IIα or -β protein levels and Topo-II activity, cleavable complex formation or cell cycle parameters. Recently, Martinchick et al (1997) found that Topo-IIα protein levels (as determined by immunohistochemistry) were increased in malignant ovarian neoplasms when compared with borderline tumours.

The aim of the present study is to evaluate the regulation of Topo-llα and β levels in untreated ovarian cancers in more detail. Especially for Topo-IIβ, only very limited data in human tumours (and especially for ovarian cancer) are available. Topo-II mRNA levels were determined by Northern blotting for Topo-IIα and -β, and with a quantitative reverse transcriptase polymerase chain reaction (RT-PCR) assay for Topo-IIα. The latter assay enables determination of Topo-IIα mRNA levels quantitatively in small tumour samples, and was used for the first time to determine Topo-IIα mRNA levels in patient material. Additionally, Topo-II protein levels were measured with Western blotting (Topo-IIα and -β) and Topo-II activity was determined with the decatenation assay.

*Received 24 December 1997*
*Revised 23 July 1998*
*Accepted 29 July 1998*

*Correspondence to:* EGE de Vries, Division of Medical Oncology, Department of Internal Medicine, University Hospital, PO Box 30.001, 9700 RB Groningen, The Netherlands

### MATERIALS AND METHODS

### Human material

Tumour specimens were obtained from patients with untreated epithelial ovarian adenocarcinomas operated consecutively at co-

**Table 1** Clinicopathological characteristics of 15 ovarian carcinoma samples

| Tumour | TVI | Tumour stage | Tumour grade | Tumour histology |
|---|---|---|---|---|
| 1 | 85 | IV | II | sa |
| 2 | 95 | III | II | sa |
| 3 | 85 | III | II | ma |
| 4 | 80 | IIc | II | sa |
| 5 | 80 | IV | III | sa |
| 6 | 75 | III | III | ac |
| 7 | 70 | III | II | sa |
| 8 | 65 | III | III | sa |
| 9 | 60 | III | III | sa |
| 10 | 50 | III | III | sa |
| 11 | 40 | III | I | sa |
| 12 | 30 | III | III | sa |
| 13 | 25 | III | II | sa |
| 14 | 20 | III | II | sa |
| 15 | n.e. | III | III | ac |

Histopathological type: ac, adenocarcinoma; sa, serous adenocarcinoma; ma, mucinous adenocarcinoma; n.e., not evaluated.

**Table 2** Results obtained for 15 ovarian tumours. Shown are mRNA and protein values and overall Topo-II activity in these tumours. Tumour 1 was defined as the 100% value for each assay

| Tumour (TVI) | mRNA | | Protein | | Activity | RT-PCR |
|---|---|---|---|---|---|---|
| | Topo-IIα | Topo-IIβ | Topo-IIα | Topo-IIβ | Topo-II | Topo-IIα |
| 1 (85) | 100% | 100% | 100% | 100% | 100% | 100% |
| 2 (95) | 13 | 140 | 24 | 82 | 100 | 25 |
| 3 (85) | 52 | 191 | 58 | 74 | 133 | 80 |
| 4 (80) | 52 | 65 | 72 | 20 | 30 | 60 |
| 5 (80) | 30 | 65 | 20 | 8 | 24 | n.m.[a] |
| 6 (75) | 9 | 102 | 46 | 11 | 48 | 59 |
| 7 (70) | 61 | 149 | 27 | 69 | 183 | 80 |
| 8 (65) | 9 | 37 | 16 | 26 | 24 | n.m. |
| 9 (60) | 43 | 140 | 48 | 37 | 100 | n.m. |
| 10 (50) | 43 | 102 | 51 | 56 | 48 | 52 |
| 11 (40) | 9 | 60 | 7 | n.d.[b] | 133 | n.m. |
| 12 (30) | 61 | 167 | 15 | 3 | 133 | n.m. |
| 13 (25) | 17 | 107 | 10 | 7 | 30 | n.m. |
| 14 (20) | 117 | 107 | 109 | 34 | n.d. | n.m. |
| 15 (n.e.[c]) | 26 | 60 | 1 | n.d. | 14 | n.m. |

[a]n.m., not measured; [b]n.d., not detectable; [c]n.e., not evaluated.

operating hospitals in the northern part of the Netherlands in 1994. Tumour collection was supervised by a pathologist. One part of the tumour was embedded in paraffin for routine histology and the other part was stored in liquid nitrogen at –180°C. For Topo-II assays, the last sample was fractioned in liquid nitrogen and pulverized and homogenized mechanically using a microdismembranator at 0°C. The powder was distributed in equal portions for isolation of RNA and protein extracts.

### Patient characteristics

All patients were staged according to the International Federation of Obstetrics and Gynaecology (FIGO) classification. Tumours were histologically classified according to the World Health Organization (WHO) classification using paraffin-embedded tissue sections (Serov et al, 1973). Carcinomas were graded into well- (I), moderately (II), and poorly differentiated (III) adenocarcinomas (Sobre et al, 1982). Acetone fixated 4-μm tumour sections from the frozen material were stained with haematoxylin and eosin (HE) to determine the tumour volume index (TVI; percentage of malignant tissue in tumour specimen) of the samples. The TVI was measured by a point counting technique, using a 42-point grid placed on a projection microscope ×200 as described by Baak et al (1988).

### Isolation of total RNA

Total RNA was isolated using a guanidine isothiocyanate/caesium chloride method (Chirgwin et al, 1979). The quality of all RNA samples was checked on formaldehyde-containing agarose gels. Only RNA samples with clearly visible ribosomal bands were used for Northern blotting, RT-PCR, Western blotting and Topo-II activity experiments.

### Northern blot analysis

RNA was vacuum slotblotted onto positively charged nylon membranes (Hybond N+, Amersham, UK). Probes were labelled with [$^{32}$P]dCTP (3000 Ci mmol$^{-1}$, Amersham) using an oligo-labelling kit (Pharmacia, Woerden, The Netherlands). The Topo-IIα probe SP1 was kindly provided by KB Tan (Chung et al, 1989). The Topo-IIβ probe was derived by PCR from a plasmid containing the entire Topo-IIβ cDNA sequence (pCDM8; provided by ID Hickson). Briefly, the Topo-IIβ-specific PCR primers are positioned at bases 3675–3694 (5′-GGAAGAGACAATGCCCTCAC-3′), and 4756–4776 (5′-CAGAGAAGGTGGCTCAGTAGG-3′) [numbering according to the EMBL DNA library (access number X68060; see Chung et al (1989) for sequence comparisons]. Blots were hybridized overnight at 65°C in 0.5 M disodium hydrogen phosphate, pH 7.2, 1 mM EDTA, 7% sodium dodecyl sulphate (SDS). Post-hybridization washes were performed in 2 × SSC (1 × SSC = 0.15 M sodium chloride, 0.015 M sodium citrate, pH 7.0)/0.1% SDS, 1 × SSC/0.1% SDS and 0.1 × SSC/0.1% SDS respectively, at 65°C for 30 min. Membranes were exposed for at least 5 h to Kodak X-Omat XAR radiographic film between intensifying screens at –80°C.

Band intensities were quantified using the LKB Ultroscan XL laser densitometer (Pharmacia). The blots were stripped and rehybridized with a 28S rRNA probe, after which the signals were corrected for 28S rRNA band intensities.

### Topo-IIα RT-PCR

After Northern blotting, only from seven of the ten tumours with a TVI ≥ 50% was RNA available for Topo-IIα RT-PCR. The Topo-IIα RT-PCR assay was performed as described previously (Withoff et al, 1994). Briefly, a series of different recombinant Topo-IIα RNA dilutions (ranging from 0.5 to 200 pg) was mixed with 100 ng of tumour RNA containing the unknown amount of Topo-IIα mRNA.

The recombinant Topo-IIα RNA was synthesized by in vitro transcription of a Topo-IIα sequence containing a 78-bp insert. Each mixture was converted into cDNA using reverse transcriptase (RT) and a Topo-IIα-specific PCR primer. The recombinant cDNA and the cellular Topo-IIα cDNA are co-amplified using the primer mentioned before and a second Topo-IIα-specific primer.

© Cancer Research Campaign 1999

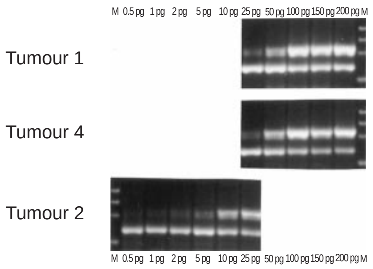

**Figure 1** Representative Topo-IIα RT-PCR results showing tumour 1, 4 and 2 displaying high, intermediate and low Topo-IIα mRNA levels. The range of recombinant RNA used is shown above the photographs. The recombinant PCR product is 430 bp long; the Topo-IIα PCR product 342 bp. The intermediate band is the result of a rec-cDNA/Topo-IIα-cDNA hybridization (see Withoff et al, 1994). M symbolizes the DNA marker (molecular weight of the bands from top to bottom: 517 bp, 453 bp, 394 bp and 298 bp)

During this reaction, the recombinant and the tumour Topo-IIα mRNA compete for the primers present. After PCR, the reaction products can be separated on an agarose gel electrophoretically. The signal ratio between the two PCR products is determined by densitometry. In the lane in which the ratio is 1.0, the amount of tumour Topo-IIα mRNA molecules present must have been equal to the known amount of recombinant RNA which was added. Taking into account the lengths of the recRNA and the Topo-IIα mRNA, one can calculate the corresponding amount of Topo-IIα mRNA in the tumour sample.

### Isolation of extracts for Western blot and activity assay purposes

Protein extracts were isolated from powdered tumour material as previously described, using a 0.35 M sodium chloride buffer (Van der Zee et al, 1991). Total protein concentration was determined in a fraction of the extract with the Lowry assay (Lowry et al, 1951). For Western blotting purposes, part of the extract was diluted 1:1 in standard Western blot sample buffer (0.5 M tris-HCl, pH 6.8, 4% SDS, 20% glycerol, 0.002% bromophenol blue, 10% 2-mercapto-ethanol) and stored at –80°C. The other half of the extract was diluted 1:1 in 87% glycerol for the Topo-II activity assay.

### Topo-IIα and β Western blotting

Protein (50 or 100 μg) was size fractionated by sodium dodecyl sulphate polyacrylamide gel electrophoresis (SDS-PAGE) (7.5%) according to the procedure of Laemmli (1970) and blotted onto PVDF membranes (Millipore, Etten-Leur, the Netherlands) using a semidry blot system. Topo-IIα protein levels were determined with a polyclonal antibody (Cambridge Research Biochemicals, Northwich, Cheshire, UK) using the Western-Light chemiluminescent detection system (Tropix, Bedford, MA, USA). Topo-IIβ levels were measured with a polyclonal rabbit antibody that has been extensively described in literature (Boege et al, 1995; Meyer et al, 1997) and that was kindly provided by Dr F Boege (Würzburg, Germany). Binding of this antibody was detected with the ECL-chemiluminescence kit (Amersham, Little Chalfont, UK). Chemiluminescence signals were visualized on Kodak X-Omat XAR radiographic film. Quantitation was performed by scanning autoradiographs with a LKB Ultroscan XL laser densitometer (Pharmacia).

As a positive control, 5 μg of $GLC_4$ (a well-defined human small-cell lung cancer cell line with known Topo-II mRNA and protein levels; Versantvoort et al, 1995) protein extract was used in each blot.

### Topo-II activity assay

Topo-II catalytic activity assay was performed by measurement of decatenation of kDNA in the presence of 1 mM ATP as described previously (De Jong et al, 1990). Nuclear extract of the well-defined small-cell lung carcinoma cell line $GLC_4$ was taken as a positive control for each assay (Zijlstra et al, 1987).

### Statistics

One-tailed Spearman rank correlations were computed (SPSS) to analyse correlations ($r$) between mRNA and protein levels for each isozyme.

Additionally, correlations between isozyme protein levels and Topo-II activity were calculated. Only tumour samples with a TVI ≥ 50% were used for these purposes. For seven of the tumours with a TVI ≥ 50%, correlations were computed between Topo-IIα RT-PCR results and Topo-IIα Northern blot data, and between Topo-IIα RT-PCR values and Topo-IIα protein levels. Additionally, two-tailed Spearman rank correlations were calculated between the TVI and Topo-II isozyme protein levels and between the Topo-IIβ/Topo-IIα ratio and Topo-II activity. Only $P$-values less than 0.05 were considered to be significant.

### RESULTS

### Patient characteristics

In Table 1, the clinicopathological characteristics of the 15 tumour samples are presented. Ten tumours had a TVI ≥ 50%. One tumour was classified stage I–II and 14 tumours stage III–IV, according to the FIGO classification.

### Topo-IIα and β mRNA levels

Northern slot blotting was performed as described above (the films are not shown). Tumour 1 was used as an internal standard. The signals were scanned, the Topo-II/28S ratio was calculated and the results were expressed as a percentage of the 100% value which was given to tumour 1. In Table 2, the data obtained for all tumours is summarized. The mean Topo-IIα mRNA level was 43%, with a range from 9% to 117%. The mean Topo-IIβ mRNA value was 106% (range 37–191%).

### Topo-IIα quantitative RT-PCR

Three representative RT-PCR results are shown in Figure 1. The results of seven tumours are presented in Table 2 and show a large variability (mean value 65%, range 25–100%), just as was found for the Northern blot data.

### Topo-IIα and β protein levels and Topo-II activity

In Figure 2, representative Topo-IIβ Western blot results are shown (Topo-IIα blots have been shown previously; Van der Zee et al, 1994*a*). In all the samples with detectable Topo-IIβ protein levels, we detected the band at 180 kDa.

*© Cancer Research Campaign 1999*

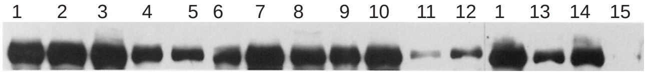

**Figure 2** Topo-IIβ Western blot results showing the tumours described in Tables 1 and 2 (numbering of the tumours as in these tables)

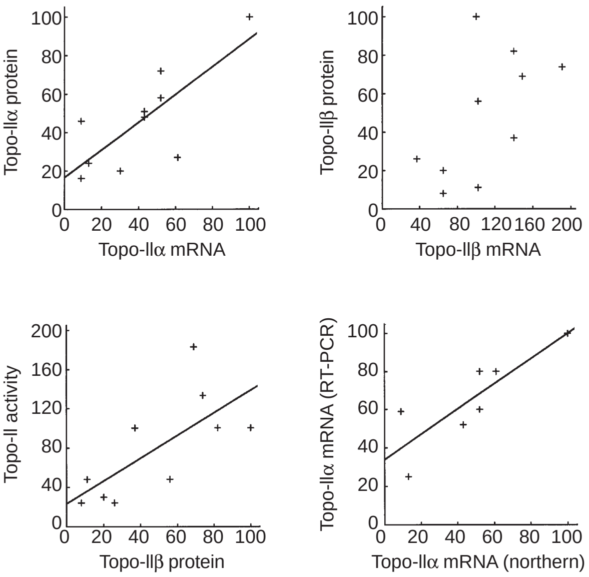

**Figure 3** This figure shows some of the correlations described in the Results section graphically. Only the results found for tumours with a TVI ≥ 50% are shown

After scanning the signals densitometrically, the results were expressed as a percentage of the value obtained for tumour 1. The results are also shown in Table 2. Topo-IIα protein levels range from 1% to 109%, with a mean value of 40%. The mean Topo-IIβ protein level was 40% (range 3–100%). Topo-II activities were also expressed relative to the result obtained for tumour 1. The results are summarized in the last column of Table 2. Using the decatenation assay, varying Topo-II activities were found (mean 79%, range 14–183%).

### Correlations between several parameters

The results obtained for the tumours with a TVI ≥ 50% were used to search for correlations between the results of the various assays. In Figure 3, some of the relevant data are shown graphically. Topo-IIα mRNA levels correlated with Topo-IIα protein level ($r$ = 0.72; $P$ = 0.01) (Figure 3A), and there was an almost significant correlation between Topo-IIβ mRNA level and Topo-IIβ protein level ($r$ = 0.53; $P$ = 0.06) (Figure 3B).

No correlation was found between Topo-IIα protein level and Topo-II activity ($r$ = 0.35; $P$ = 0.16), however the correlation between Topo-IIβ protein level and Topo-II activity was found to be significant ($r$ = 0.74; $P$ < 0.01) (Figure 3C). The ratio Topo-IIβ/Topo-IIα did not correlate with Topo-II activity ($r$ = 0.33; $P$ = 0.350). TVI and Topo-IIα or -β protein levels did not correlate for these ten tumours. Additionally, correlations were found between the Topo-IIα RT-PCR data and the Topo-IIα Northern blot results ($r$ = 0.83; $P$ = 0.01) (Figure 3D), and between the Topo-IIα RT-PCR values and Topo-IIα protein levels ($r$ = 0.68; $P$ = 0.047).

No correlation was found between any clinicopathological factor and Topo-IIα or -β protein levels nor with Topo-II activity levels.

### Topo-IIα and -β protein levels in non-malignant ovarian cystadenomas

As Topo-IIβ appeared to be an important contributor to overall Topo-II activity in malignant ovarian tissues, it was decided to measure Topo-IIα, but more importantly Topo-IIβ protein levels, in eight non-malignant ovarian cystadenomas as well. Previously, it was shown that Topo-II activity is lower in cystadenoma than in malignant tissue (Van der Zee et al, 1991). In the eight cystadenoma samples, no Topo-IIα could be detected, and only three of the eight samples showed detectable Topo-IIβ protein bands on Western blot (results not shown).

### DISCUSSION

The first studies on Topo-II levels in human cancers were performed in haematological malignancies (e.g. Gekeler et al, 1992; Ishikawa et al, 1993; Beck et al, 1994; Kaufmann et al, 1994; Larsson et al, 1994; McKenna et al, 1994). However, recently more information has become available on Topo-II levels in solid tumours or in primary cultures of solid tumours (e.g. Kim et al, 1991; Van der Zee et al, 1991, 1994*a,b*; Larsson et al, 1994; Cornarotti et al, 1996; Davies et al, 1996; Sandri et al, 1996; Martinchick et al, 1997; Turley et al, 1997). The measured levels have been correlated with clinical response to Topo-II drugs with varying results.

Possible explanations for the discrepancy between Topo-II levels and response to chemotherapy in solid tumours might be that in most studies only one Topo-II parameter was determined (for instance mRNA level) or the fact that more often only Topo-IIα levels have been studied, thereby overlooking a possible role for Topo-IIβ. Additionally, in solid tumours, most cells are in $G_0/G_1$ phase in which Topo-IIα levels are very low (Kimura et al, 1994). The possible role of the more recently discovered Topo-IIβ isoenzyme, which is expressed throughout the cell cycle at approximately the same level and which contributes differently (Topo-IIβ is more processive) to overall Topo-II activity (Drake et al, 1989), may therefore be underestimated. The possible importance of Topo-IIβ in tumours was shown by data obtained from chronic lymphocytic leukaemias in which Topo-IIα mRNA levels were often low or even undetectable, whereas Topo-IIβ levels were relatively high (Beck et al, 1994).

Previously, it was observed that in untreated human ovarian carcinomas heterogeneity exists between tumours regarding their Topo-II activity levels. No relation was found between Topo-IIα or -β protein levels with Topo-II activity, cleavable complex formation or cell cycle parameters by Van der Zee et al (1991, 1994*a*). In contrast, Martinchick et al (1997) did find a correlation between Topo-IIα protein levels and the cell cycle marker MIB1.

In the present study, Topo-IIα and -β levels were analysed in untreated ovarian tumours (mRNA, protein and activity). Topo-IIα and -β mRNA and protein levels could be detected in practically all tumours.

Considerable variability was already found on mRNA level (see

© Cancer Research Campaign 1999

Table 2), which may result from differences in transcriptional regulation or from gene dosage effects.

For the quantification of Topo-IIα mRNA, a very sensitive RT-PCR assay has become available recently (Withoff et al, 1994). This assay was used to determine Topo-IIα mRNA levels in some of the samples in order to validate the feasibility of using this assay on tumour material. The results obtained with this assay correlate with Topo-IIα Northern and Western blot results. These findings indicate that the RT-PCR assay is a powerful tool for determining Topo-IIα mRNA levels in patient samples quantitatively, and may be advantageous when only small amounts of tumour are available.

The Topo-IIα Northern blotting data correlated significantly with Topo-IIα Western blot results, indicating that both techniques may be used to quantify Topo-IIα levels. For Topo-IIβ mRNA, and protein levels, an almost significant correlation was found.

These results are in contrast with results obtained for six breast cancer cell lines, in which no correlation between mRNA and protein level could be found for neither Topo-IIα nor Topo-IIβ (Houlbrook et al, 1995). Whether these differences are due to differences in tumour type (it was shown that Topo-IIα and Topo-IIβ mRNA levels vary in different tumour types; D'Andrea et al, 1995) or to the fact that Houlbrook et al (1995) studied cell lines, whereas in the present study tumours are examined, is unclear. A possible relationship between the expression of each isozyme and Topo-II activity was not investigated by Houlbrook et al (1995).

In the ovarian tumour samples measured in this study, Topo-IIβ protein levels correlate significantly with Topo-II activity levels, whereas Topo-IIα protein levels do not. This finding is in agreement with the assumption that in solid tumours, in which most cells are in $G_0/G_1$ phase, Topo-IIα levels are low. Topo-IIβ levels seem to be stable during different phases of the cell cycle. The results suggest that Topo-IIβ may present a target for chemotherapy of ovarian cancer. However, a possible contribution of Topo-IIα to Topo-II activity cannot be ruled out completely, especially because the possibility of post-translational modifications of both isozymes has not been investigated in this study. Additionally, Martinchick et al (1997) have shown that certain tumours contain a high percentage of Topo-IIα-positive cells. However, the relationship between Topo-IIα staining and Topo-II activity was not investigated by these authors. The results obtained for the eight cystadenomas suggest that Topo-IIα as well as Topo-IIβ protein levels are up-regulated in most ovarian cancer tissues when compared with non-malignant ovarian cystadenomas.

On the contrary, Cornarotti et al (1996) showed that Topo-IIα mRNA levels were increased in malignant over benign or normal ovarian tissues, but Topo-IIβ mRNA levels were not. However, these authors mentioned that comparison between malignant and benign or normal tissue was troubled by the fact that the majority of their non-malignant samples consisted of stromal tissue. Cornarotti et al (1996) also measured only one Topo-II parameter (mRNA, but not protein levels nor overall Topo-II activity).

When Topo-IIβ contributes importantly to Topo-II activity in ovarian cancer development, Topo-IIβ-specific chemotherapy might be useful in treatment of this type of cancer. The possible importance of Topo-IIβ as an anti-cancer drug target was also suggested by Sandri et al (1996) and Turley et al (1997), who described that the β-isoform is more widely expressed than the α-isoform in several non-ovarian tumour types. At present, no Topo-IIβ-specific drugs are known, although dramatic decreases in Topo-IIβ levels seem to contribute to mitoxantrone resistance in several resistant cell lines (Harker et al, 1995; Withoff et al, 1996).

### ACKNOWLEDGEMENTS

We would like to thank M Krans and GJ Meersma for expert technical assistance. This study was supported by grant 91-12 of the Dutch Cancer Society.

### REFERENCES

Baak JPA, Schipper NW, Wisse-Brekelmans ECM, Ceelen Th, Bosman FT, van Geuns H and Wils J (1988) The prognostic value of morphometric features and cellular DNA content in cisplatin treated late ovarian cancer patients. *Br J Cancer* **57**: 503–508

Beck J, Niethammer D and Gekeler V (1994) High mdr1- and mrp-, but low topoisomerase IIα-gene expression in B-cell chronic lymphocytic leukaemias. *Cancer Lett* **86**: 135–142

Boege F, Andersen A, Jensen S, Zeidler R and Kreipe H (1995) Proliferation-associated nuclear antigen Ki-S1 is identical with topoisomerase IIα. Delineation of a carboxyterminal epitope with peptide antibodies. *Am J Pathol* **146**: 1302–1308

Cannistra SA (1993) Cancer of the ovary. *N Engl J Med* **329**: 1550–1559

Chirgwin JM, Przybyla AE, MacDonald RJ and Rutter WJ (1979) Isolation of biologically active ribonucleic acid from sources enriched in ribonuclease. *Biochemistry* **18**: 5294–5299

Chung TDY, Drake FH, Tan KB, Per SR, Crooke ST and Mirabelli CK (1989) Characterization and immunological identification of cDNA clones encoding two human DNA topoisomerase II isozymes. *Proc Natl Acad Sci USA* **86**: 9431–9435

Cornarotti M, Capranico G, Bohm S, Oriana S, Spatti GB, Mariani L, Ballabio G and Zunino F (1996) Gene expression of DNA topoisomerase I, IIα and IIβ and response to cisplatin-based chemotherapy in advanced ovarian carcinoma. *Int J Cancer* **67**: 479–484

D'Andrea MR, Multhaupt HAD, Farber PA and Fogleson PD (1995) Expression of genes for DNA topoisomerase IIα and DNA topoisomerase IIβ in human tumours. *Proc Am Assoc Cancer Res* **36**: 2690

Davies SL, Popert R, Coptcoat M, Hickson ID and Masters JRW (1996) Response to epirubicin in patients with superficial bladder cancer and expression of the topoisomerase II α and β genes. *Int J Cancer* **65**: 63–66

De Jong S, Zijlstra JG, De Vries EGE and Mulder NH (1990) Reduced DNA topoisomerase II activity and drug-induced DNA cleavage activity in an adriamycin-resistant human small cell lung carcinoma cell line. *Cancer Res* **50**: 304–309

Drake FH, Hofmann GA, Bartus HF, Mattern MR, Crooke ST and Mirabelli CK (1989) Biochemical and pharmacological properties of p170 and p180 forms of topoisomerase II. *Biochemistry* **28**: 8154–8160

Gekeler V, Frese G, Noller A, Handgretinger R, Wilisch A, Schmidt H, Muller CP, Dopfer R, Klingebiel T, Diddens H, Probst H and Niethammer D (1992) MDR1/P-glycoprotein, topoisomerase and glutathione-S-transferase pi gene expression in primary and relapsed state adult and childhood leukaemias. *Br J Cancer* **66**: 507–517

Harker WG, Slade DL, Parr RL, Feldhoff PW, Sullivan DM and Holguin MH (1995) Alterations in the topoisomerase IIα gene, messenger RNA, and subcellular protein distribution as well as reduced expression of the DNA topoisomerase IIβ enzyme in a mitoxantrone-resistant HL-60 human leukemia cell line. *Cancer Res* **55**: 1707–1716

Houlbrook S, Addison CM, Davies SL, Carmichael J, Stratford IJ, Harris AL and Hickson ID (1995) Relationship between expression of topoisomerase II isoforms and intrinsic sensitivity to topoisomerase II inhibitors in breast cancer cell lines. *Br J Cancer* **72**: 1454–1461

Ishikawa H, Kawano MM, Okada K, Tanaka H, Tanabe O, Sakai A, Asaoku H, Iwato K, Nobuyoshi M and Kuramoto A (1993) Expressions of DNA topoisomerase I and II gene and the genes possibly related to drug resistance in human myeloma cells. *Br J Haematol* **83**: 68–74

Kaufmann SH, Karp JE, Jones RJ, Miller CB, Schneider E, Zwelling LA, Cowan K, Wendel K and Burke PJ (1994) Topoisomerase II levels and drug sensitivity in adult acute myelogenous leukemia. *Blood* **83**: 517–530

© Cancer Research Campaign 1999

Kim R, Hirabayashi N, Nishiyama M, Saeki S, Toge T and Okada K (1991) Expression of MDR1, GST-π and topoisomerase II as an indicator of clinical response to adriamycin. *Anticancer Res* **11**: 429–432

Kimura K, Saijo M, Ui M and Enomoto T (1994) Growth state- and cell cycle-dependent fluctuation in the expression of two forms of DNA topoisomerase II and possible specific modification of the higher molecular weight form in the M phase. *J Biol Chem* **269**: 1173–1176

Laemmli UK (1970) Cleavage of structural proteins during the assembly of the head of bacteriophage T4. *Nature (London)* **227**: 680–685

Larsson R and Nygren P (1994) Cytotoxic activity of topoisomerase II inhibitors in primary cultures of tumor cells from patients with human hematologic and solid tumors. *Cancer* **74**: 2857–2862

Lowry OH, Rosebrough NJ, Farr AL and Randall RJ (1951) Protein measurement with the folin–phenol reagent. *J Biol Chem* **193**: 265–275

Martinchick JC, Rahn MP, Jolles CJ and Holden JA (1997) Human DNA topoisomerase II-α: immunohistochemical staining for enzyme protein in tumors of ovarian origin. *Int J Oncol* **10**: 1229–1234

McKenna SL, West RR, Whittaker JA, Padua RA and Holmes JA (1994) Topoisomerase II α expression in acute myeloid leukaemia and its relationship to clinical outcome. *Leukemia* **8**: 1498–1502

Meyer KN, Kjeldsen E, Straub T, Knudsen BR, Hickson ID, Kikuchi A, Kreipe H and Boege F (1997) Cell cycle-coupled relocation of types I and II topoisomerases and modulation of catalytic enzyme activities. *J Cell Biol* **136**: 775–788

Pommier Y (1993) DNA topoisomerase I and II in cancer chemotherapy: update and perspectives. *Cancer Chemother Pharmacol* **32**: 103–108

Sandri MI, Hochhauser D, Ayton P, Camplejohn RC, Whitehouse R, Turley H, Gatter K, Hickson ID and Harris AL (1996) Differential expression of the topoisomerase IIα and β genes in human breast cancers. *Br J Cancer* **73**: 1518–1524

Serov SF, Scully RE and Sobin LH (1973) *Histological Typing of Ovarian Carcinomas*, pp. 17–18. World Health Organization: Geneva

Sobre B, Frankendal B and Veress B (1982) Importance of histological grade in the prognosis of epithelial ovarian carcinomas. *Obstet Gynecol* **59**: 567–573

Trimble EL, Arbuck SG and McGuire WP (1994) Options for primary chemotherapy of epithelial ovarian cancer: taxanes. *Gynecol Oncol* **55**: S114–S121

Turley H, Comley M, Houlbrook S, Nozaki N, Kikuchi A, Hickson ID, Gatter K and Harris AL (1997) The distribution and expression of the two isoforms of DNA topoisomerase II in normal and neoplastic human tissues. *Br J Cancer* **75**: 1340–1346

Van der Zee AGJ, Hollema H, De Jong S, Boonstra H, Gouw A, Willemse PHB, Zijlstra JG and De Vries EGE (1991) P-glycoprotein expression and DNA topoisomerase I and II activity in benign tumors of the ovary and in malignant tumors of the ovary, before and after platinum/cyclophosphamide chemotherapy. *Cancer Res* **51**: 5915–5920

Van der Zee AGJ, De Jong S, Keith WN, Hollema H, Boonstra H and De Vries EGE (1994*a*) Quantitative and qualitative aspects of topoisomerase I and IIα and β in untreated and platinum/cyclophosphamide treated malignant ovarian tumors. *Cancer Res* **54**: 749–755

Van der Zee AGJ, De Vries EGE, Hollema H, Kaye SB, Brown R and Keith WN (1994*b*) Molecular analysis of the topoisomerase IIα gene and its expression in human ovarian cancer. *Ann Oncol* **5**: 75–81

Van der Zee AGJ, Hollema HH, De Bruijn HW, Willemse PHB, Boonstra H, Mulder NH, Aalders JG and De Vries EGE (1995) Cell biological markers of drug resistance in ovarian carcinoma. *Gynecol Oncol* **58**: 165–178

Versantvoort CHM, Withoff S, Broxterman HJ, Kuiper CM, Scheper RJ, Mulder NH and De Vries EGE (1995) Resistance associated factors in human small cell lung carcinoma $GLC_4$ sublines with increasing adriamycin resistance. *Int J Cancer* **61**: 375–380

Withoff S, Smit EF, Meersma GJ, Van den Berg A, Timmer-Bosscha H, Kok K, Postmus PE, Mulder NH, De Vries EGE and Buys CHCM (1994) Quantitation of DNA topoisomerase IIα messenger ribonucleic acid levels in a small cell lung cancer cell line and two drug resistant sublines using a polymerase chain reaction-aided transcript titration assay. *Lab Invest* **71**: 61–66

Withoff S, De Vries EGE, Keith WN, Nienhuis EF, Van der Graaf WTA, Uges DRA and Mulder NH (1996) Differential expression of DNA topoisomerase IIα and -β in P-gp and MRP-negative VM26, mAMSA and mitoxantrone-resistant sublines of the human SCLC cell line $GLC_4$. *Br J Cancer* **74**: 1869–1876

Zijlstra JG, De Vries EGE and Mulder NH (1987) Multifactorial drug resistance in an adriamycin-resistant human small cell lung carcinoma cell line. *Cancer Res* **47**: 1780–1784

*© Cancer Research Campaign 1999*
